# Improving the quality of the chicken fillet using chitosan, gelatin, and starch coatings incorporated with bitter orange peel extract during refrigeration

**DOI:** 10.1002/fsn3.3432

**Published:** 2023-05-15

**Authors:** Maryam Azizkhani, Sara Kavosi, Razieh Partovi

**Affiliations:** ^1^ Department of Food Hygiene, Faculty of Veterinary Medicine Amol University of Special Modern Technologies Amol Iran

**Keywords:** chicken fillet, chitosan, citrus, gelatin, starch

## Abstract

The preserving potential of biopolymer coatings can be improved by adding natural antimicrobial and antioxidant compounds. The objective of this study was to evaluate the effect of natural coatings (gelatin (Gel), chitosan (Ch), and modified starch (MS)) incorporated with bitter orange peel extract (BOE) on the quality of the chicken fillets during cold. BOE had a high amount of phenolic compounds (145.28 mgGAE/g). Coating the fillets with pure BOE exerted a higher inhibitory effect against bacterial growth compared to composite coatings without extract. Lower microbial count (2–3 log CFU/g on days 9 and 12 of storage) was observed in the samples coated with composite biopolymers incorporated with BOE in comparison to the coatings without BOE. Composite coatings of Gel/MS/BOE showed lower FFA in the fillets followed by Gel/Ch/BOE and MS/Ch/BOE. The lowest TVB‐N belonged to MS/Ch/BOE followed by Gel/Ch/BOE and Gel/MS/BOE which were 17.05, 17.39, and 19.40 mg/100 g at the end of the storage. Among the samples, pure BOE, Gel/MS/BOE, Gel/Ch/BOE, and MS/Ch/BOE showed the lowest peroxide value and the coatings containing chitosan had a slower rate of hydroperoxide generation. Drip loss showed a descending trend in all coated samples except for an enhancement in control and BOE‐coated fillets, 6.42% and 6.39%, respectively, on day 12 of storage. Samples coated with Gel/MS and Gel/MS/BOE had the lowest drip loss during the storage period (5.96% and 5.98%, respectively). It should be noted that coatings containing chitosan had higher antimicrobial and antioxidant effects. The effect of the coatings as antimicrobial barriers and preservative agents were as follows: Gel/Ch/BOE > MS/Ch/BOE > Gel/MS/BOE. It can be concluded that the applied composite coatings in this work have a high potential to maintain and improve the quality of raw chicken fillets during storage in the refrigerator.

## Graphical abstract

The effect of natural coatings (gelatin (Gel), chitosan (Ch), and modified starch (MS)) incorporated with bitter orange peel extract (BOE) on the quality of the chicken fillets during cold storage was evaluated. The effect of the coatings as antimicrobial barriers and preservative agents were as follows: Gel/Ch/BOE > MS/Ch/BOE > Gel/MS/BOE. It can be concluded that the applied composite coatings in this work have a high potential to maintain and improve the quality of raw chicken fillets during storage in the refrigerator.

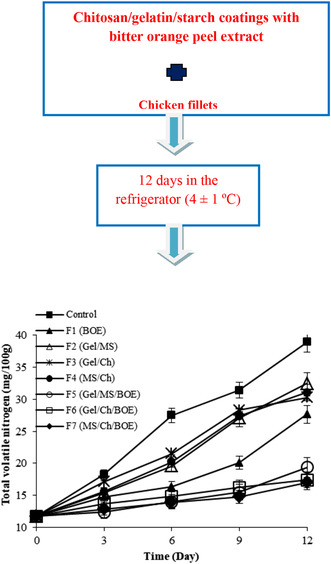

## 1 INTRODUCTION

In recent years, along with the advancement of technology in the food packaging industry, the use of edible biodegradable coatings and films has been developed to maintain the quality of the products. These edible coatings can be prepared from a variety of biopolymer compounds, including proteins, polysaccharides, fibers, and lipids which are found abundantly in nature (Ng & Kurisawa, 2021). These biopolymers possess the potential of creating an obstacle against the penetration of water molecules, oxygen, the release of compounds responsible for aroma and flavor, slow down the oxidation process, reduce moisture loss and weight loss, and prevent the occurrence of surface microbial contamination. Also, these types of coatings have a positive role in maintaining the sensory characteristics and texture quality as well as increasing the shelf life of the product. Foodstuffs such as fish, shrimp, fruit, and vegetables are packed using these biopolymer coatings in pure or combined (composite) form and sometimes incorporated with preservative compounds such as essential oils and herbal extracts (Smaoui et al., 2022). These new approaches are used in combination with other food preservation methods such as cooling (in the refrigerator) and freezing (in the freezer) to store products (Khwaldia et al., 2010).

Chitosan is a biopolymer and linear polysaccharide consisting of glucosamine molecules obtained from the deacetylation of chitin. Due to its inherent antimicrobial properties and high potential to form a film, it is used extensively in food packaging (Nowzari et al., 2013). Gelatin is a protein derivative that is formed from the partial hydrolysis of collagen. It consists of glycine, proline, and 4‐hydroxyproline units. Gelatin powder is transparent, colorless, and almost tasteless. Depending on the process used, two types of gelatin are obtained, namely, type A (of acid hydrolysis) and type B (of alkaline hydrolysis). Gelatin is one of the most common food coatings and is used in the preservation of various meat products due to its high ability to form a film, high water‐binding capacity, availability, and low price (Gedarawatte et al., 2021). It has been reported that composite coatings containing chitosan and gelatin used in food preservation had a very good mechanical performance. This property has been attributed to the formation of polyelectrolyte complexes through electrostatic interactions between protonated amine groups of chitosan and negatively charged side substituents in gelatin or collagen (e.g., carboxylate groups; Pereda et al., 2011).

Starch is a polysaccharide macromolecule consisting of glucose units. Starch‐based coatings are biodegradable, low‐cost, and environmentally friendly (Suhag et al., 2020). However, due to being hydrophilic, these coatings are weak barriers to the penetration of water molecules. To solve this problem, starch is modified by thermal, chemical, or enzymatic methods. Modified starch has a higher capacity to retain water and resistance to shrinkage. In several studies, the use of modified starch with other polymers such as gelatin and essential oils or plant extracts with antimicrobial effects has been reported as a suitable technique for food preservation (Kumar et al., 2021; Suhag et al., 2020).

In research studying the effect of chitosan‐gelatin coatings on the rate of oxidative acceleration in rainbow trout fillets during storage in the refrigerator (4 ± 1°C), the results showed that chitosan‐gelatin coatings maintained the quality characteristics of fish fillets, controlled the bacterial population and also increased the shelf life of the samples (Nowzari et al., 2013). In another study, a composite edible coating containing chitosan and starch was used to minimize weight loss and delay microbial spoilage in blackberries. The data showed that the composite edible coating of chitosan and starch (1:1) was effective in minimizing weight loss and mold decay. The researchers announced that the use of a combined edible coating of chitosan and starch is a suitable method to prevent moldy rot and increase the shelf life of black berries in cold storage conditions (Ojeda et al., 2021). Also, it is reported that a mixture of gelatin (10%) and chitosan (1%) sprayed on beef as a coating exerted antioxidant and antimicrobial effects and increased the shelf life of the product (Gedarawatte et al., 2021).

The preservative activity of edible coatings can be increased by adding natural antimicrobial and antioxidant compounds. By‐products of the citrus processing industry are naturally rich sources of phenolic compounds. The peel, which makes up about half of the fruit's mass, contains the highest amount of flavonoids in citrus. Extracts obtained from the peel of citrus fruits such as orange, bitter orange, lemon, lime, and mandarin have a high potential as antioxidants in preventing oxidative reactions (Zou et al., 2016). Raw food products such as chicken fillet due to its high content of unsaturated fatty acids (omega 3), high protein and free amino acids content, and considerable moisture are prone to adverse microbial, biochemical, and tissue changes during the distribution, sale, and storage which limits the shelf life of the product (Ahmadi et al., 2020). Bitter orange is one of the most cultivated citrus in north of Iran and its peel is considered agricultural waste but is proved to contain phenolic compounds (Zou et al., 2016). As phenolic compounds possess antioxidant and antimicrobial potential and there is no study about incorporating bitter orange peel extract in biopolymers (to be used as natural preservative), we aimed to evaluate the antioxidant and antimicrobial effect of biopolymer coating (containing gelatin, chitosan, and modified starch) incorporated with phenolic compounds of bitter orange peel on the chicken fillets during the storage period in the refrigerator.

## 2 MATERIALS AND METHODS

### 2.1 Chemicals and materials

All the reagents, chemicals, and biopolymers were purchased from Scharlau Chemical Co. Bitter orange (*Citrus aurantium*) was purchased from the local market of Sari (Mazandaran province, north of Iran) and peeled using a kitchen knife. Two hundred grams of the peels was cut into pieces of 2–2.5 cm^2^ and kept at −34 ± 1°C to inhibit the oxidation of the bioactive compounds. Four kilograms of fresh chicken fillets (fresh carcass transferred from slaughterhouse to market as chilled and cut into fillets just before purchasing in the market) was obtained from a local supermarket in Sari (Iran) and transferred to the laboratory in an ice box.

### 2.2 Preparing peel extract

The peel extract was prepared according to the method explained by Marzouk (2013). Briefly, the peel pieces were added to the aqueous ethanol (80% v/v) with the solvent ratio of 1:20 (1 g of peels in 20 mL of ethanol) and homogenized with a blender (MCL‐HX‐4GM, MeCan Co.). An ultrasound‐assisted extraction technique was applied to increase the extraction efficiency. The homogenized peel samples were transferred to a volumetric flask and placed in an ultrasonic bath (S30H model, Alma Co.). The sonication was carried out at a power of 150 W, 40°C for 30 min. The sonicated extracts were filtered using Whatman paper No. 1 under a vacuum. Then, the extracts were mixed with n‐hexane and incubated in the ultrasonic bath for 20 min at room temperature to remove the oily components. The ethanol phase was separated from the oily phase and centrifuged (RF 10000 model, Orum Tajhiz Gostar Co.) at 11,000 *g* for 10 min at room temperature followed by concentrating with a rotary evaporator (RV10 V‐C model, Azin Co.) at 50°C (Marzouk, 2013).

### 2.3 Total phenolic content

The total phenolic content (TPC) of the peel extract was measured through Folin–Ciocalteu method. The results were reported as mg gallic acid equivalents (GAE) per gram (g) of the peel (Sir Elkhatim et al., 2018).

### 2.4 Preparation of composite coatings containing bitter orange peel extract

Modified starch was prepared by thermal heating (physical process) from a commercial source (Alborz Starch Co.), the gelatin type B (Halal gelatin of bovine bone produced through an alkaline process) was kindly donated by Iran Capsule Co., and chitosan (medium molecular weight, viscosity 300–800 cP) was purchased from Scharlau Chemical Co. Modified starch (MS) solution (30% w/v in distilled water), gelatin (Gel) solution (1% w/v in distilled water), and chitosan (Ch) solution (1% w/v in 1% acetic acid) were prepared. All solutions were separately homogenized using a magnetic stirrer (Hei‐PLATE Mix 20 I, Heidolph Instruments Co.) at room temperature (25 ± 1°C) for 1 h. Then, the 50:50 ratio of the solutions was mixed and homogenized at 10,000 *g* for 10 min applying a homogenizer (model HC‐2A, Hoacheng Co.). In the next step, 1% (v/v) of glycerol as the plasticizer and 5% (v/v) bitter orange peel extract (BOE) were added to the above solutions to prepare the coating formula. The prepared coating solutions were mixed and stirred for 1 h applying a magnetic stirrer (Gedarawatte et al., 2021).

### 2.5 Applying the coatings on the chicken fillets

Chicken fillets were rinsed with cold distilled water for 3 min and the outer surface was dried with a dryer paper. The fillets were cut into 50 ± 5 g pieces in a sterile condition and divided into eight groups: Control, F1 (BOE), F2 (Gel/MS), F3 (Gel/Ch), F4 (MS/Ch), F5 (Gel/MS/BOE), F6 (Gel/Ch/BOE), and F7 (MS/Ch/BOE). The samples were soaked in their individual coating solutions for 30 s, then taken out and placed on a sterile sieve (for 2 min) to drain. The dipping and drying steps were repeated twice. The control group included chicken fillet pieces dipped in deionized water. The samples were allowed to drain for 5 min in a biological safety cabinet, placed in polyethylene zip‐locker bags, and stored in the refrigerator (4 ± 1°C). The chemical, microbial, and sensory evaluations were carried out at 3‐day intervals during 12‐day storage time.

### 2.6 pH determination

To measure pH value, 10 g of the fillet samples was homogenized with 25 mL of neutral distilled water, incubated at room temperature for 10 min, and filtered using Whatman paper no. 1. The pH of the filtrate was determined using a pH meter (Wideman et al., 2016).

### 2.7 Determination of drip loss

Drip loss is water escaping from raw meat during storage. Drip loss was measured by suspending individually 50‐g chicken fillet samples in polyethylene bags without any touch with the sides of the bags, for 24 h at 4°C. Then, samples were removed from the bags, gently dried, and weighed. Drip loss was determined as the percentage of weight lost (Rahman et al., 2015).

$$\text{Drip loss}\;\left(\%\right):\left[\left(\text{sample weight}\;\left(g\right)-\text{sample weight after}\;24\;h\;\left(g\right)\right)/\text{sample weight}\;\left(g\right)\right]\times 100.$$

### 2.8 Texture analysis

The sample preparation for texture analysis was carried out by taking raw fillet pieces with a circular shape using a cylindrical punch with 3 cm long and 3 cm diameter. The texture of the fillet samples was analyzed for firmness by peak shear force (*g*) applying a texture analyzer instrument (TA.XTplusC, Stable Microsystems Co.) using a 2 mm rectangular steel probe. Texture softness/firmness was determined by the shear energy (*N* × mm). Ten fillet samples (*n* = 10) of each treatment were tested and the readings were carried out in triplicate (Khan et al., 2022).

### 2.9 Microbial analyses

To prepare decimal dilutions of fillet samples, 10 g of each sample was homogenized with 90 mL of 0.1% peptone water using a stomacher (BagMixer 400 SW, HealthCare Technologies Co.) at 4 *g* for 60 s, and inoculated onto the plate count agar (for total mesophilic and psychrotrophic bacteria), violet red bile glucose agar (for Enterobacteriaceae family), de Man Rogosa and Sharpe agar (for lactic acid bacteria), and Pseudomonas agar (for *Pseudomonas* spp.). The incubation condition was 37°C for 1 day for mesophilic, 7°C for 10 days for psychrotrophic, 37°C for 1 day for *Pseudomonas* spp., 30°C for 2 days for lactic acid bacteria, and 37°C for 1 day for Enterobacteriaceae count. All counts were carried out in triplicate and expressed as log_10_ CFU/g (Shahbazi et al., 2016).

### 2.10 Chemical analysis

#### 2.10.1 Measuring free fatty acids (FFA) content

Free fatty acids value was determined according to the method of Rahman et al. (2015) and reported on basis of grams of oleic acid in 100 g of the sample (Rahman et al., 2015).

#### 2.10.2 Measuring total volatile base nitrogen (TVB‐N)

To determine TVB‐N, the method explained by Jonaidi Jafari was used (Jonaidi Jafari et al., 2018).

#### 2.10.3 Measuring peroxide value (PV)

The peroxide value was determined according to the method explained by Rahman et al. (2015) and was expressed as milliequivalent peroxide per kilogram of the sample (Rahman et al., 2015).

### 2.11 Statistical analysis

All the experiments were carried out in triplicate. Data analysis was carried out using Two‐way analysis of variance (ANOVA) and Duncan's Test in SPSS software version 22.00. Significant differences between the means of each characteristic for the different coatings were determined by Tukey's HSD test. The statistically significant differences between mean values were determined at confidence levels of 99% and 95%.

## 3 RESULTS AND DISCUSSION

### 3.1 pH

The results of pH measurement in chicken fillets treated with biopolymer coatings are presented in Table 1. There were significant differences between the samples coated with pure peel extract (F1) and the samples coated with extract‐incorporated biopolymers and the control (*p* < .01). F3 (Gel/Ch) and F7 (MS/Ch/BOE) showed pH values similar to the control on the 3rd day of the storage (*p* > .01). As seen, in all treatments, there was a significant decrease in pH value toward the end of the storage time (*p* < .01). The decrease in pH of treatments, especially those containing BOE, is due to the presence of organic compounds such as organic acids and the conversion of glycogen of the muscle to lactic acid in cease of oxygen supply. Also, during storage in the refrigerator, a slight decrease in pH may occur due to the formation of carbonic acid from the CO_2_ resulting from the metabolism of spoilage‐causing microorganisms (Chmiel et al., 2018). The lowest final pH value was found in F1 (BOE) and F2 (Gel/MS) and F5 (Gel/MS/BOE) and the highest pH was observed in the control, F3 (Gel/Ch), and F4 (MS/Ch) (*p* < .01). It seems that among the treatments, those including chitosan had higher pH values at the end of the storage time (*p* < .01). We assumed that the decrease in pH in chicken fillets could be due to the growth of LAB during storage which is also claimed by other studies (Clarke et al., 2017; Gedarawatte et al., 2021). This is similar to the findings of Gedarawatte et al. (2021) who observed chitosan‐coated beef had higher pH values throughout the storage time. This could be attributed to the significant reduction of LAB counts achieved in chitosan‐coated samples compared to gelatin‐coated samples (Gedarawatte et al., 2021). The samples coated with starch‐contained composites, particularly in combination with gelatin, showed lower pH which may be due to the trivial degradation of starch and is used as a substrate for lactic acid generation (Tkaczewska et al., 2022).

**TABLE 1:** pH of the chicken fillets treated with bitter orange extract and biocomposite coatings during storage at 4 ± 1°C.

	Time (day)				
	0	3	6	9	12
Control	5.92 ± 0.20^Aa^ *	5.90 ± 0.17^Aa^	5.85 ± 0.09^Bb^	5.79 ± 0.45^Cc^	5.76 ± 0.31^Dc^
F1 (BOE)	5.92 ± 0.20^Aa^	5.86 ± 0.10^Bc^	5.80 ± 0.15^Cc^	5.73 ± 0.85^De^	5.69 ± 0.25^Ee^
F2 (Gel/MS)	5.92 ± 0.20^Aa^	5.87 ± 0.48^Bb^	5.80 ± 0.10^Cc^	5.75 ± 0.62^Dd^	5.68 ± 0.40^Ee^
F3 (Gel/Ch)	5.92 ± 0.20^Aa^	5.90 ± 0.38^Aa^	5.88 ± 0.43^Aa^	5.87 ± 0.15^Ba^	5.86 ± 0.31^Ba^
F4 (MS/Ch)	5.92 ± 0.20^Aa^	5.89 ± 0.10^Aa^	5.86 ± 0.57^Bb^	5.85 ± 0.16^Bb^	5.83 ± 0.24^Cb^
F5 (Gel/MS/BOE)	5.92 ± 0.20^Aa^	5.84 ± 0.73^Bd^	5.77 ± 0.50^Cd^	5.71 ± 0.38^Df^	5.67 ± 0.49^Ef^
F6 (Gel/Ch/BOE)	5.92 ± 0.20^Aa^	5.88 ± 0.28^Ab^	5.81 ± 0.13^Bc^	5.74 ± 0.10^Ce^	5.70 ± 0.22^Dd^
F7 (MS/Ch/BOE)	5.92 ± 0.20^Aa^	5.89 ± 0.47^Aa^	5.80 ± 0.64^Bc^	5.76 ± 0.44^Cd^	5.71 ± 0.85^Dd^

* Different letters in the columns (a–e) and in the rows (A–E) indicate a statistically significant difference (*p* < .05). Data are presented as mean ± SD.

### 3.2 Drip loss

According to Table 2, the drip loss of the fillet samples on the first day was approximately 6.12%–6.16% (Table 2). Drip loss showed a descending trend in all coated samples except for an enhancement in control and F1 (BOE‐coated fillets), 6.42% and 6.39%, respectively, on day 12 of storage (*p* < .01). Samples coated with Gel/MS and Gel/MS/BOE had the lowest drip loss during the storage period (5.96% and 5.98%, respectively, on day 12; *p* < .01). The drip loss of the fillet samples coated with biopolymer composite was considerably lower than the control (without coating) and F1 (BOE coated without biopolymers) (*p* ˂ .05). Also, the drip loss of biopolymer‐coated fillets on the 12th day was significantly lower than those on the 3, 6, and 9th day of refrigerated storage (*p* ˂ .05). We found that coatings containing modified starch showed a higher potential for holding moisture in the tissue compared to gelatin and chitosan; also, it should be noted that gelatin had a better effect on reducing drip loss than chitosan in composites (*p* < .01). The results of Li et al. (2018) demonstrated that the quality of Nile tilapia fillets was improved by the coating of chitosan and/or Jicama starch. Compared with pure chitosan coating, composite coating of chitosan/Jicama starch showed a stronger effect on reducing the drip loss that expressed the higher ability of starch molecules to hold water inside the polymer structure (Li et al., 2018). Wei et al. (2019) declared that gelatin (0%, 2.5%, and 5%) reduced the drip loss of golden pompano (*Trachinotus blochii*) fillets stored at 4°C over 14 days (Wei et al., 2019). In this work, the chicken fillets coated with starch, gelatin, and chitosan reflected significantly lower drip loss compared to BOE‐coated samples and control that indicates higher muscle integrity and water holding of the texture (Yang et al., 2018). On the other hand, microbial activity leads to the hydrolysis of collagens and myofibrillar proteins on poultry meat surface and the release of intercellular fluids during storage (Roslan et al., 2019). The lower drip loss shows lower microbial activity in biopolymer‐coated fillets.

**TABLE 2:** Drip loss (%w/w) of the chicken fillets treated with bitter orange extract and biocomposite coatings during storage at 4 ± 1°C.

	Time (day)				
	0	3	6	9	12
Control	6.14 ± 0.10^Da^ *	6.11 ± 0.27^Db^	6.25 ± 0.22^Ca^	6.33 ± 0.50^Ba^	6.42 ± 0.42^Aa^
F1 (BOE)	6.16 ± 0.48^Ca^	6.21 ± 0.17^Ba^	6.26 ± 0.65^Ba^	6.27 ± 0.75^Bb^	6.39 ± 0.13^Aa^
F2 (Gel/MS)	6.13 ± 0.20^Aa^	6.13 ± 0.85^Ab^	6.10 ± 0.19^Ab^	6.09 ± 0.32^Ac^	5.96 ± 0.73^Be^
F3 (Gel/Ch)	6.15 ± 0.72^Aa^	6.12 ± 0.33^Ab^	6.08 ± 0.70^Bb^	6.03 ± 0.18^Bd^	6.01 ± 0.29^Bd^
F4 (MS/Ch)	6.12 ± 0.97^Aa^	6.10 ± 0.45^Ab^	6.10 ± 0.39^Ab^	6.09 ± 0.56^Ac^	6.08 ± 0.41^Ab^
F5 (Gel/MS/BOE)	6.12 ± 0.50^Aa^	6.08 ± 0.65^Ac^	6.05 ± 0.51^Ac^	5.96 ± 0.82^Be^	5.98 ± 0.50^Be^
F6 (Gel/Ch/BOE)	6.15 ± 0.81^Aa^	6.09 ± 0.88^Bc^	6.05 ± 0.28^Bc^	6.02 ± 0.20^Bd^	6.02 ± 0.92^Bc^
F7 (MS/Ch/BOE)	6.14 ± 0.70^Aa^	6.07 ± 0.35^Bc^	6.06 ± 0.14^Bc^	6.03 ± 0.36^Bd^	6.02 ± 0.35^Bc^

* Different letters in the columns (a–e) and in the rows (A–E) indicate a statistically significant difference (*p* < .05). Data are presented as mean ± SD.

### 3.3 Texture

The texture and structure of meat are considered important freshness properties that depend on several parameters such as firmness, springiness, chewiness, and also the internal cross‐linking of the connective tissue and the detachment of muscle fibers (Cheng et al., 2014). The mean texture firmness of the fillets on day 0 was 4.02 ± 0.10 N which gradually decreased in all the fillet samples during storage (*p* < .05; Table 3). The fillet samples coated with F1 (coated with BOE) and composite biopolymers (F2–F7) showed significantly lower (*p* ˂ .05) firmness on days 6, 9, and 12 of storage compared to the control. It is found that composite coatings incorporated with BOE (F5, F6, and F7) provided higher texture softness (*p* < .05) in chicken fillets in comparison to composite coatings without BOE (F2, F3, and F4), as seen in Table 3. The results indicated that BOE, in the form of free extract or incorporated in composite coating, decreased the texture firmness. Also, the samples treated with a combination of Gel and Ch expressed lower firmness followed by MS/Ch (*p* < .05). The highest firmness belonged to Gel/MS and Gel/MS/BOE‐treated samples (*p* < .05). In a study by Li et al. (2018) on the applying chitosan and starch coating on fish fillets during cold storage, the hardness value decreased gradually during storage which could be due to the activity of bacteria and the decomposition of myofibrillar tissue. This value was higher in chitosan and starch composite‐coated samples than those in the other groups. It seems that biopolymers provide a barrier against microbial activity and tissue degradation (Li et al., 2018).

**TABLE 3:** Texture firmness (*N*) of the chicken fillets treated with bitter orange extract and biocomposite coatings during storage at 4 ± 1°C.

	Time (day)				
	0	3	6	9	12
Control	4.02 ± 0.25^Ca^ *	4.10 ± 0.19^AC^	4.06 ± 0.28^Be^	4.04 ± 0.41^Be^	3.90 ± 0.83^De^
F1 (BOE)	4.02 ± 0.25^Aa^	3.92 ± 0.31^Bd^	3.85 ± 0.39^Cf^	3.77 ± 0.45^Df^	3.72 ± 0.16^Ef^
F2 (Gel/MS)	4.02 ± 0.25^Ea^	4.28 ± 0.46^Da^	4.39 ± 0.80^Cb^	4.57 ± 0.20^Bb^	4.95 ± 0.44^Aa^
F3 (Gel/Ch)	4.02 ± 0.25^Ca^	4.15 ± 0.55^Bb^	4.17 ± 0.72^Bd^	4.21 ± 0.13^Ad^	4.25 ± 0.36^Ad^
F4 (MS/Ch)	4.02 ± 0.25^Da^	4.19 ± 0.30^Cb^	4.21 ± 0.44^Cc^	4.33 ± 0.68^Bc^	4.42 ± 0.50^Ac^
F5 (Gel/MS/BOE)	4.02 ± 0.25^Ea^	4.27 ± 0.42^Da^	4.50 ± 0.85^Ca^	4.65 ± 0.22^Ba^	4.86 ± 0.75^Ab^
F6 (Gel/Ch/BOE)	4.02 ± 0.25^Da^	4.11 ± 0.17^Cc^	4.20 ± 0.35^Bc^	4.24 ± 0.11^Bd^	4.30 ± 0.16^Ad^
F7 (MS/Ch/BOE)	4.02 ± 0.25^Ea^	4.13 ± 0.61^Dc^	4.25 ± 0.29^Cc^	4.35 ± 0.87^Bc^	4.47 ± 0.10^Ac^

* Different letters in the columns (a–e) and in the rows (A–E) indicate a statistically significant difference (*p* < .05). Data are presented as mean ± SD.

### 3.4 Total phenolic content

The industries of food and agricultural products generate considerable amounts of byproducts rich in phenolic compounds that are valuable natural sources of antioxidants and antimicrobials such as polyphenols. In sweat and bitter oranges, the peel accounts for almost 30% of the fruit mass which contains the highest concentration of flavonoids in comparison to the other parts of the fruit. In this study, we quantified the peel TPC in ethanolic extract of bitter orange peel as 145.28 mg GAE/g. It is reported that polymethoxylated flavonoids and flavonoid C‐ and O‐glycosides are the most abundant flavonoids (Haggag et al., 1999; Xi et al., 2017). According to Sawalha et al. (2009), naringin and neohesperidin are the major polyphenols in bitter orange peels (Sawalha et al., 2009).

### 3.5 Microbiological evaluation

#### 3.5.1 Total viable count

The population of total viable bacterial (TVC) in chicken fillets is presented in Table 1. In the control group, TVC increased during storage and its value reached 7.75 on day 12 (Table 4). TVC of coated fillet samples incorporated with biopolymers and BOE (F5, F6, and F7) was lower compared to the other groups (*p* < .05). In the F6 (Gel/Ch/BOE) and F7 (MS/Ch/BOE) groups, the count was 4.76 and 4.69 log CFU/g on day 12, respectively, which showed a significant difference from the other treatments (*p* < .05). As seen in Table 1, the total population of viable bacteria was as follows: control > F2 > F4 > F3 > F1 > F5 > F6 > F7. Coating the fillet samples with pure extract exerted a higher inhibitory effect against bacterial growth compared to composite coatings without extract (F2, F3, and F4; *p* < .05). Lower TVC (2–3 log CFU/g on days 9 and 12 of storage) was observed in the samples coated with composite biopolymer coatings incorporated with BOE in comparison to the coatings without BOE (*p* < .05). It seems that there is a synergistic effect between biopolymers and BOE in inhibiting microbial growth. The same results were reported by Mehdizadeh et al. (2018) in TVC of beef coated with edible MS/Ch composite film incorporated with *Thymus kotschyanus* essential oil and *Punica granatum* peel extracts during 21‐day storage at 4°C (Mehdizadeh et al., 2018). The findings of a study by Hassan et al. (2022) indicated that the growth of mesophilic bacteria in the chicken breast fillets treated with Ch/Gel/papaya leaves and Ch/Gel/thyme extract was considerably slower than control and the samples coated with Ch/Gel (Hassan et al., 2022). Also, an edible coating of Gel/starch incorporated with cucumber peel extract and cumin essential oil inhibited the growth of mesophilic aerobic bacteria in a cheese model (Esparvarini et al., 2022). It is well‐established that the application of biopolymer coatings fortified with essential oils or extracts could induce the structural destruction of mitochondrial and cellular membrane lipids in the bacteria and inhibit microbial proliferation. This antimicrobial effect is attributed to the phenolic compounds and terpenoids that are present in bitter orange peel extract. As declared by previous studies, biopolymers act as a barrier against oxygen transfer which results in the growth inhibition of aerobic bacteria (Song et al., 2011).

**TABLE 4:** Total viable count (log CFU/g) of the chicken fillets treated with bitter orange extract and biocomposite coatings during storage at 4 ± 1°C.

	Time (day)				
	0	3	6	9	12
Control	1.85 ± 0.07^Aa^ *	3.55 ± 0.16^Ba^	4.18 ± 0.35^Ca^	5.86 ± 0.48^Da^	7.75 ± 0.22^Ea^
F1 (BOE)	1.85 ± 0.07^Aa^	2.11 ± 0.36^Bf^	3.72 ± 0.25^Cc^	4.49 ± 0.10^Dd^	5.25 ± 0.17^Ec^
F2 (Gel/MS)	1.85 ± 0.07^Aa^	3.27 ± 0.08^Bb^	4.16 ± 0.51^Ca^	5.81 ± 0.44^Da^	7.63 ± 0.38^Ea^
F3 (Gel/Ch)	1.85 ± 0.07^Aa^	2.87 ± 0.15^Bc^	3.90 ± 0.27^Cb^	5.25 ± 0.10^Db^	6.31 ± 0.59^Eb^
F4 (MS/Ch)	1.85 ± 0.07^Aa^	2.69 ± 0.16^Bd^	3.95 ± 0.46^Cb^	5.36 ± 0.31^Db^	6.40 ± 0.55^Eb^
F5 (Gel/MS/BOE)	1.85 ± 0.07^Aa^	2.86 ± 0.19^Bc^	3.35 ± 0.21^Cd^	4.79 ± 0.37^Dc^	5.02 ± 0.41^Ed^
F6 (Gel/Ch/BOE)	1.85 ± 0.07^Aa^	2.30 ± 0.05^Be^	3.15 ± 0.28^Ce^	4.19 ± 0.56^De^	4.76 ± 0.39^Ee^
F7 (MS/Ch/BOE)	1.85 ± 0.07^Aa^	2.23 ± 0.35^Be^	3.01 ± 0.10^Ce^	4.14 ± 0.50^De^	4.69 ± 0.15^Ee^

* Different letters in the columns (a–e) and in the rows (A–E) indicate a statistically significant difference (*p* < .05). Data are presented as mean ± SD.

#### 3.5.2 Psychrotrophic bacteria

The dominant group of microorganisms that causes spoilage in fresh meat, poultry, and fish products during cold storage is psychrotrophic (Saenz‐García et al., 2020). As presented in Table 5, on days 9 and 12, the population of psychrotrophic in chicken fillets coated with biopolymer composite incorporated with BOE was about 1.1–1.3 log CFU/g lower than the samples treated with biopolymers and about 3 log CFU/g lower than control indicating that F5, F6, and F7 were effective against psychrotrophic bacteria (*p* < .05). It seems that the phenolic compounds in BOE can act as antibacterial agents, had a synergistic effect with biopolymers, and caused disruptions in microbial cells which led to a decrease in the growth rate. It should be noted that samples treated with Gel/Ch/BOE had the lowest psychrotrophic population during the storage period followed by MS/Ch/BOE with 0.15 log CFU/g difference (*p* < .05). During cold storage, the psychrotrophic count of the samples treated with pure BOE was 3.5 log CFU/g lower than the samples coated with biopolymer composite (without BOE; *p* < .05). The composite coatings containing chitosan showed higher activity against the growth of psychrotrophic bacteria (*p* < .05). In a study by Nowzari et al. (2013), the psychrotrophic count in the trout fillets coated with chitosan and gelatin was about 6.5 log CFU/g on day 16 of cold storage with a significant difference compared to control and samples coated with pure gelatin or pure chitosan (Nowzari et al., 2013). It is reported that chitosan is an effective antimicrobial agent (Pereda et al., 2011) which is attributed to the presence of the amino groups with a positive charge and their interaction with negatively charged molecules on the bacterial cell membrane, resulting in the leakage of intracellular constituents of the bacteria. Pereda et al. (2011) announced the inhibitory effect of gelatin coating against the growth of *listeria monoccytogenes* which is likely related to the presence of oligopeptide chains (with side‐chain amino groups) resulting from hydrolysis of gelatin (Pereda et al., 2011).

**TABLE 5:** Psychrotrophic bacteria count (log CFU/g) of the chicken fillets treated with bitter orange extract and biocomposite coatings during storage at 4 ± 1°C.

	Time (day)				
	0	3	6	9	12
Control	2.91 ± 0.08^Aa^ *	4.83 ± 0.21^Ba^	5.71 ± 0.33^Ca^	6.55 ± 0.10^Da^	7.85 ± 0.13^Ea^
F1 (BOE)	2.91 ± 0.08^Aa^	3.17 ± 0.43^Bc^	3.81 ± 0.20^Cd^	4.07 ± 0.25^Dd^	4.38 ± 0.26^Ed^
F2 (Gel/MS)	2.91 ± 0.08^Aa^	3.67 ± 0.25^Bb^	4.50 ± 0.61^Cb^	4.93 ± 0.18^Db^	5.25 ± 0.19^Eb^
F3 (Gel/Ch)	2.91 ± 0.08^Aa^	3.11 ± 0.29^Bc^	4.33 ± 0.10^Cc^	4.81 ± 0.35^Db^	5.01 ± 0.42^Ec^
F4 (MS/Ch)	2.91 ± 0.08^Aa^	3.13 ± 0.11^Bc^	4.25 ± 0.48^Cc^	4.75 ± 0.51^Dc^	5.00 ± 0.22^Ec^
F5 (Gel/MS/BOE)	2.91 ± 0.08^Aa^	3.00 ± 0.26^Bd^	3.25 ± 0.16^Ce^	3.82 ± 0.36^De^	4.14 ± 0.10^Ee^
F6 (Gel/Ch/BOE)	2.91 ± 0.08^Aa^	3.10 ± 0.10^Bc^	3.27 ± 0.09^Ce^	3.65 ± 0.31^Df^	3.85 ± 0.19^Ef^
F7 (MS/Ch/BOE)	2.91 ± 0.08^Aa^	3.10 ± 0.07^Bc^	3.20 ± 0.21^Be^	3.50 ± 0.10^Cg^	3.70 ± 0.25^Dg^

* Different letters in the columns (a–e) and in the rows (A–E) indicate a statistically significant difference (*p* < .05). Data are presented as mean ± SD.

Vásconez et al. (2009) found that chitosan coating reduced the growth rate of mesophilic and psychrotrophic bacteria but the chitosan/starch had lower antibacterial activity. The same results were observed in our study which could be due to the interaction between chitosan and starch in which starch reduces the antimicrobial effect of chitosan (Vásconez et al., 2009).

#### 3.5.3 *Pseudomonas* spp.

It is obvious from the results of Table 6 that composite coatings (without BOE) could not act efficiently in reducing the growth rate of *Pseudomonas* spp. The samples treated with BOE and biopolymers incorporated with BOE had significantly lower *Pseudomonas* spp. population (about 1 log CFU/g) compared to other samples during cold storage (*p* < .05). The most effective coatings in inhibiting the growth of *Pseudomonas* spp. were F5 (Gel/MS/BOE), F6 (Gel/Ch/BOE), and F7 (MS/Ch/BOE; *p* < .05). Also, F1 (BOE) showed significantly higher antibacterial activity compared to the control and F2–F4 (*p* < .05). The antimicrobial activity of the bitter orange extract is attributed to phenolic compounds, tannins, saponins, and flavonoids that are biologically active. It has been reported that tannin in citrus peel extracts forms irreversible bonds with proline‐rich proteins leading to the inhibition of protein synthesis in the cells (Shimada, 2006). Our data indicated that the coatings consisted of chitosan had higher effect against *Pseudomonas* growth. The mechanism of antibacterial action of chitosan may be due to the disruption of the lipopolysaccharide layer of the cellular outer membrane in gram‐negative bacteria; on the other hand, chitosan can act as a barrier against oxygen and moisture transfer (Pereda et al., 2011).

**TABLE 6:** *Pseudomonas* Spp. count (log CFU/g) of the chicken fillets treated with bitter orange extract and biocomposite coatings during storage at 4 ± 1°C.

	Time (day)				
	0	3	6	9	12
Control	2.10 ± 0.25^Aa^ *	3.05 ± 0.25^Ba^	3.76 ± 0.21^Ca^	4.38 ± 0.00^Da^	4.89 ± 0.30^Ea^
F1 (BOE)	2.10 ± 0.25^Aa^	2.90 ± 0.10^Bb^	3.20 ± 0.15^Cc^	3.52 ± 0.05^Dc^	3.81 ± 0.26^Ed^
F2 (Gel/MS)	2.10 ± 0.25^Aa^	2.95 ± 0.17^Bb^	3.40 ± 0.35^Cb^	3.78 ± 0.15^Db^	4.47 ± 0.29^Eb^
F3 (Gel/Ch)	2.10 ± 0.25^Aa^	2.54 ± 0.00^Bc^	3.22 ± 0.20^Cc^	3.68 ± 0.13^Db^	4.19 ± 0.18^Ec^
F4 (MS/Ch)	2.10 ± 0.25^Aa^	2.65 ± 0.30^Bc^	3.43 ± 0.41^Cb^	3.70 ± 0.21^Db^	4.25 ± 0.10^Ec^
F5 (Gel/MS/BOE)	2.10 ± 0.25^Aa^	2.55 ± 0.14^Bc^	2.96 ± 0.00^Cd^	3.28 ± 0.16^Dd^	3.61 ± 0.08^Ee^
F6 (Gel/Ch/BOE)	2.10 ± 0.25^Aa^	2.20 ± 0.35^Bd^	2.39 ± 0.25^Ce^	2.65 ± 0.40^Df^	3.00 ± 0.34^Ef^
F7 (MS/Ch/BOE)	2.10 ± 0.25^Aa^	2.15 ± 0.50^Bd^	2.45 ± 0.11^Ce^	2.80 ± 0.00^De^	3.08 ± 0.10^Ef^

* Different letters in the columns (a–e) and in the rows (A–E) indicate a statistically significant difference (*p* < .05). Data are presented as mean ± SD.

#### 3.5.4 Enterobacteriaceae count

The population of Enterobacteriaceae in the samples during cold storage is presented in Table 7. The results indicated the inhibitory effects of the coatings on the bacterial growth rate with a significant difference compared to the control (*p* < .05). The highest antimicrobial activity against the growth of Enterobacteriaceae was observed for F6 (Gel/Ch/BOE) followed by F7 (MS/Ch/BOE), F5 (Gel/MS/BOE), and F1 (BOE) which showed a considerable difference in comparison to other treatments (*p* < .05). The composite treatments containing chitosan had a higher antibacterial effect as found for F6 and F7 (*p* < .05). The antibacterial effect of the chitosan‐based coatings incorporated with polyphenols of lemon peel extract in raw poultry meat is reported by previous studies. The fillets coated with chitosan in combination with lemon peel extract showed longer bacterial lag phases and growth inhibition (Maru et al., 2021). Moreno et al. (2018) found that Gel/starch composite coating incorporated with lauroyl‐l‐arginine ethyl ester significantly extended the shelf life of chicken breast fillets during 20‐day of cold storage and decreased total coliform and *Escherichia coli* count compared to the control (Moreno et al., 2018).

**TABLE 7:** Enterobacteriaceae count (log CFU/g) of the chicken fillets treated with bitter orange extract and biocomposite coatings during storage at 4 ± 1°C.

	Time (day)				
	0	3	6	9	12
Control	1.26 ± 0.10^Aa^ *	2.74 ± 0.11^Ba^	3.50 ± 0.25^Ca^	4.61 ± 0.18^Da^	5.58 ± 0.21^Ea^
F1 (BOE)	1.26 ± 0.10^Aa^	2.04 ± 0.19^Bd^	2.69 ± 0.10^Cd^	3.23 ± 0.03^Dd^	3.63 ± 0.11^Ed^
F2 (Gel/MS)	1.26 ± 0.10^Aa^	2.50 ± 0.10^Bb^	3.27 ± 0.14^Cb^	4.25 ± 0.39^Db^	4.83 ± 0.55^Eb^
F3 (Gel/Ch)	1.26 ± 0.10^Aa^	2.18 ± 0.33^Bc^	3.00 ± 0.48^Cc^	4.15 ± 0.09^Dc^	4.49 ± 0.37^Ec^
F4 (MS/Ch)	1.26 ± 0.10^Aa^	2.20 ± 0.06^Bc^	3.09 ± 0.36^Cc^	4.13 ± 0.10^Dc^	4.45 ± 0.20^Ec^
F5 (Gel/MS/BOE)	1.26 ± 0.10^Aa^	1.65 ± 0.18^Be^	2.05 ± 0.14^Cg^	2.81 ± 0.25^De^	3.46 ± 0.37^Ee^
F6 (Gel/Ch/BOE)	1.26 ± 0.10^Aa^	1.50 ± 0.23^Bf^	2.28 ± 0.05^e^	2.65 ± 0.08^Df^	2.80 ± 0.31^Ef^
F7 (MS/Ch/BOE)	1.26 ± 0.10^Aa^	1.45 ± 0.00^Bf^	2.40 ± 0.11^Cf^	2.69 ± 0.15^Df^	2.88 ± 0.16^Ef^

* Different letters in the columns (a–e) and in the rows (A–E) indicate a statistically significant difference (*p* < .05). Data are presented as mean ± SD.

It is known that alkaloid compounds and flavonoids of the ethanolic extract of citrus peel possess an extensive range of biological activities such as cytostatic, antimicrobial, and antioxidant in addition to toxicity against foreign cells and organisms. Also, terpenoids in ethanolic extracts are involved in cell membrane degradation through the disruption of lipophilic compounds (Kademi & Garba, 2017). We concluded that BOE and biocomposite/BOE expressed significant inhibitory effects on the growth and proliferation of Enterobacteriaceae.

#### 3.5.5 Lactic acid bacteria (LAB) count

Table 8 presents the LAB count in chicken fillets coated with bitter orange peel extract and biopolymer composites during cold storage. The initial population of LAB in fillets was 4.68 log CFU/g which increased gradually during storage until reaching about 3–5 log CFU/g on day 12. According to the obtained data, pure BOE decreased the growth of the LAB and applying biopolymers/BOE resulted in a considerable decrease in the LAB population (*p* < .05). The growth of LAB in fillets coated with Gel/Ch/BOE was significantly lower than the other samples throughout storage followed by MS/Ch/BOE and MS/Gel/BOE. Moreno et al. (2018) reported that composite coating of starch and gelatin on chicken fillets actively reduced the population of LAB which was significantly lower than the control; our results are in accordance with their findings (Moreno et al., 2018). Also, Jonaidi Jafari et al. (2018) reported the lowest LAB count in the chicken fillets treated with chitosan coatings incorporated with propolis extract compared to pure chitosan, pure propolis, and control (Jonaidi Jafari et al., 2018).

**TABLE 8:** Lactic acid bacteria count (log CFU/g) of the chicken fillets treated with bitter orange extract and biocomposite coatings during storage at 4 ± 1°C.

	Time (day)				
	0	3	6	9	12
Control	4.68 ± 0.15^Aa^ *	4.92 ± 0.25^Ba^	5.17 ± 0.10^Ca^	5.40 ± 0.37^Da^	5.75 ± 0.22^Ea^
F1 (BOE)	4.68 ± 0.15^Aa^	4.45 ± 0.09^Bc^	3.90 ± 0.23^Cd^	3.71 ± 0.27^Dc^	3.55 ± 0.16^Ed^
F2 (Gel/MS)	4.68 ± 0.15^Aa^	4.89 ± 0.07^Ba^	5.04 ± 0.10^Ca^	5.29 ± 0.12^Da^	5.26 ± 0.30^Eb^
F3 (Gel/Ch)	4.68 ± 0.15^Aa^	4.51 ± 0.18^Ab^	4.70 ± 0.23^Ab^	4.77 ± 0.19^Ab^	4.95 ± 0.61^Bc^
F4 (MS/Ch)	4.68 ± 0.15^Aa^	4.59 ± 0.10^Ab^	4.65 ± 0.17^Ab^	4.71 ± 0.26^Ab^	4.89 ± 0.14^Bc^
F5 (Gel/MS/BOE)	4.68 ± 0.15^Aa^	4.49 ± 0.33^Bc^	4.08 ± 0.26^Cc^	3.63 ± 0.11^Dc^	3.37 ± 0.21^Ed^
F6 (Gel/Ch/BOE)	4.68 ± 0.15^Aa^	4.55 ± 0.28^Ab^	4.02 ± 0.33^Bc^	3.56 ± 0.10^Cc^	3.05 ± 0.13^De^
F7 (MS/Ch/BOE)	4.68 ± 0.15^Aa^	4.31 ± 0.47^Bd^	3.78 ± 0.10^Cd^	3.64 ± 0.08^Cc^	3.09 ± 0.25^De^

* Different letters in the columns (a–e) and in the rows (A–E) indicate statistically significant difference (*p* < .05). Data are presented as mean ± SD.

### 3.6 Chemical evaluations

#### 3.6.1 Free fatty acids content

Free fatty acids (FFAs) are derived from lipid hydrolysis and cleavage of triacylglycerols due to lipase activity in high temperatures and the presence of moisture. FFAs act as pro‐oxidants and speed up the rate of hydroperoxide degradation; therefore, high FFA content in the fats causes further oxidation and results in the development of off‐flavor and unpleasant taste (Chew & Nyam, 2020). FFA may involve in the reactions with myofibrillar proteins which leads to protein aggregation (Özyurt et al., 2009). According to the data presented in Table 9, the initial FFA value of the fillet samples was around 0.2% of oleic acid and significant differences were found among the samples treated with BOE, biopolymer composite, biopolymer composites incorporated with BOE and the control (*p* < .05). A slow constant increase was observed in FFA content in coated samples throughout the storage period. This increase was significantly higher in the control compared to the treated samples (*p* < .05). The chicken fillets coated with biopolymers/BOE showed lower FFA content (0.12%–0.13%) and BOE (0.15%) compared to the samples coated with biopolymers (0.19%–0.22%) on day 12 of storage (*p* < .05). According to the results, composite coatings of Gel/MS/BOE showed lower FFA generation in the fillet samples (*p* < .05) followed by Gel/Ch/BOE and MS/Ch/BOE (*p* < .01). It was found that adding peel extract to the composite coatings caused a synergistic effect to inhibit lipolysis. The FFA content in the samples during storage was as follows: Control > Gel/MS > Gel/Ch > MS/Ch > BOE > MS/Ch/BOE > Gel/Ch/BOE > Gel/MS/BOE. Our findings are in agreement with Nowzari et al. (2013) who reported that Ch/Gel coating and film decelerated the development process of FFA formation in rainbow trout fillets during storage in refrigerator in comparison to control (Nowzari et al., 2013). Also, Kyani Haftlang et al. (2018) announced the inhibitory effect of the corn starch coating incorporated with black pepper essential oil against FFA generation in the silver carp fillet during 12‐day storage in the refrigerator (Kyani Haftlang et al., 2018). Lipases are known as surface‐active enzymes and act on their substrates at the polar/nonpolar interface. The protective effect of coatings against lipolysis may be due to prevent the moisture and oxygen penetration into the fillets tissue, slowing down the accessibility of enzymes to the substrate and thus inhibiting lipase activity (Carpen et al., 2019).

**TABLE 9:** Free fatty acid content (% oleic acid) of the chicken fillets treated with bitter orange extract and biocomposite coatings during storage at 4 ± 1°C.

	Time (day)				
	0	3	6	9	12
Control	0.02 ± 0.00^Aa^ *	0.07 ± 0.01^Ba^	0.11 ± 0.02^Ca^	0.19 ± 0.03^Da^	0.22 ± 0.04^Ea^
F1 (BOE)	0.02 ± 0.00^Aa^	0.03 ± 0.00^Ab^	0.09 ± 0.01^Bb^	0.11 ± 0.00^Bc^	0.15 ± 0.01^Cc^
F2 (Gel/MS)	0.02 ± 0.00^Aa^	0.06 ± 0.00^Ba^	0.12 ± 0.00^Ca^	0.15 ± 0.01^Db^	0.21 ± 0.03^Ea^
F3 (Gel/Ch)	0.02 ± 0.00^Aa^	0.06 ± 0.01^Bb^	0.13 ± 0.02^Ca^	0.17 ± 0.00^Da^	0.20 ± 0.05^Da^
F4 (MS/Ch)	0.02 ± 0.00^Aa^	0.05 ± 0.00^Aa^	0.12 ± 0.01^Ba^	0.17 ± 0.03^Ca^	0.19 ± 0.02^Cb^
F5 (Gel/MS/BOE)	0.02 ± 0.00^Aa^	0.04 ± 0.00^Aa^	0.05 ± 0.00^Ac^	0.10 ± 0.02^Bc^	0.12 ± 0.00^Bd^
F6 (Gel/Ch/BOE)	0.02 ± 0.00^Aa^	0.06 ± 0.00^Ba^	0.06 ± 0.00^Bc^	0.11 ± 0.02^Cc^	0.13 ± 0.02^Cc^
F7 (MS/Ch/BOE)	0.02 ± 0.00^Aa^	0.05 ± 0.01^Aa^	0.06 ± 0.00^Bc^	0.12 ± 0.01^Cb^	0.13 ± 0.00^Cc^

* Different letters in the columns (a–e) and in the rows (A–E) indicate statistically significant difference (*p* < .05). Data are presented as mean ± SD.

#### 3.6.2 Total volatile base nitrogen (TVB‐N)

Chicken fillet is a perishable product and the activity of microorganisms and endogenous enzymes leads to chemical compositional changes during storage. A common biomarker of protein and amine degradation is total volatile basic nitrogen (TVB‐N; Bekhit et al., 2021). The TVB‐N content of meat, poultry, and fish products increases during the storage period due to the degradation of proteins and other nitrogen‐containing compounds. TVB‐N accumulation occurs in parallel with chemical deterioration, microbial spoilage, considerable changes in sensory acceptability, and color and flavor changes (Bekhit et al., 2021). The data of TVB‐N for the chicken fillet samples (Table 10) revealed that in the control group, TVB‐N increased during cold storage and reached a value of 38.95 mg/100 g on day 12. In all biopolymer composite coating groups incorporated with peel extract, the TVB‐N was significantly lower compared to control and groups of biopolymers without extract or extract without biopolymers (*p* < .05) throughout the storage period. The lowest TVB‐N values belonged to F7 (MS/Ch/BOE) followed by F6 (Gel/Ch/BOE) and F5 (Gel/MS/BOE) which were 17.05, 17.39, and 19.40 mg/100 g at the end of the cold storage (*p* < .05). It must be noted that there was no significant difference between TVB‐N of F7 and F6 throughout the storage time (*p* < .05). TVB‐N of the samples coated with pure BOE was lower in comparison to the samples coated with BOE‐free‐biopolymers (*p* < .05). It should be noted that treatments containing chitosan showed lower TVB‐N compared to other samples (*p* < .05). This effect may be attributed to the inhibitory activity against bacterial growth exerted by chitosan as lower microbial population leads to lower basic nitrogen fractions (Mohan et al., 2012). In a study by Nowzari et al. (2013) on applying Gel‐Ch coating on fish fillets, TVB‐N value of the treated samples was found to be around 17 mg/100 g while for the control and reached about 25 mg/100 g at the end of the 16‐day storage period (Nowzari et al., 2013). Rajabian et al. (2019) observed lower amounts of TVB‐N in the chicken breast samples coated with potato starch incorporated with *Ziziphora clinopodioides* and *Thymus daenensis* essential oils compared to uncoated samples (Rajabian et al., 2019). It can be concluded that composite coatings incorporated with BOE are more effective than pure BOE in controlling TVB‐N of the chicken fillets.

**TABLE 10:** Total volatile nitrogen (mg/100 g) of the chicken fillets treated with bitter orange extract and biocomposite coatings during storage at 4 ± 1°C.

	Time (day)				
	0	3	6	9	12
Control	11.75 ± 0.19^Aa^ *	18.25 ± 0.11^Ba^	27.5 ± 0.32^Ca^	31.43 ± 0.29^Da^	38.95 ± 0.27^Ea^
F1 (BOE)	11.75 ± 0.19^Aa^	14.71 ± 0.20^Bb^	16.34 ± 0.20^Cb^	20.08 ± 0.34^Dc^	27.66 ± 0.31^Ec^
F2 (Gel/MS)	11.75 ± 0.19^Aa^	15.35 ± 0.08^Ba^	19.60 ± 0.15^Ca^	27.10 ± 0.17^Db^	32.50 ± 0.40^Ea^
F3 (Gel/Ch)	11.75 ± 0.19^Aa^	17.12 ± 0.10^Bb^	21.50 ± 0.22^Ca^	28.33 ± 0.30^Da^	30.27 ± 0.22^Ea^
F4 (MS/Ch)	11.75 ± 0.19^Aa^	15.60 ± 0.05^Ba^	20.25 ± 0.07^Ca^	27.35 ± 0.41^Da^	31.06 ± 0.15^Eb^
F5 (Gel/MS/BOE)	11.75 ± 0.19^Aa^	12.39 ± 0.23^Aa^	13.98 ± 0.04^Bc^	15.50 ± 0.25^Cc^	19.40 ± 0.10^Dd^
F6 (Gel/Ch/BOE)	11.75 ± 0.19^Aa^	13.75 ± 0.16^Ba^	14.85 ± 0.10^Bc^	16.22 ± 0.14^Cc^	17.39 ± 0.26^Cc^
F7 (MS/Ch/BOE)	11.75 ± 0.19^Aa^	12.85 ± 0.10^Aa^	13.92 ± 0.24^Bc^	14.76 ± 0.19^Cb^	17.05 ± 0.08^Cc^

* Different letters in the columns (a–e) and in the rows (A–E) indicate statistically significant difference (*p* < .05). Data are presented as mean ± SD.

#### 3.6.3 Peroxide value (PV)

The PV is the most common chemical test for evaluating the oxidative deterioration of fats and oils. Although hydroperoxides (as the primary products of oxidation) degrade to a mixture of volatile and nonvolatile molecules and react to the endoperoxides, the PV measurement is a useful method of monitoring oxidative deterioration changes (Gilbraith et al., 2021). The degradation of the hydroperoxides leads to the formation of a variety of secondary products such as furans, ketones, carbonyl compounds, hydrocarbons, and other compounds that are responsible for off‐flavor and rancidity in food (Kong & Singh, 2016). Hydroperoxide formation rate in the samples coated with BOE (F1) and BOE/biopolymer (F5, F6, and F7) was trivial during the first 3–6 days of the storage period (Table 11). PV of all the coated samples was found lower than control (*p* < .05) and also coatings incorporated with BOE showed lower PV than the same composites without BOE (*p* < .05). Among the samples, F1 (BOE), F5 (Gel/MS/BOE), F6 (Gel/Ch/BOE), and F7 (MS/Ch/BOE) showed the lowest hydroperoxide concentration during 12‐day storage (*p* < .05), and as it is obvious from the results that the coatings containing chitosan caused a slower rate of hydroperoxide generation in the samples. Also, during the storage period, the fillets coated with BOE expressed the same PV (0.38 meq/kg) as the fillets coated with F5 (Gel/MS/BOE) (*p* > .05) that indicates the strong antioxidant activity of phenolic compounds and flavonoids in the bitter orange peel extract against production and propagation of radicals and hydroperoxide. This study indicated that composite coatings incorporated with BOE significantly postponed the primary oxidation of the fat content of fillets, while this parameter in control increased considerably during storage. As reported by Jonaidi Jafari et al. (2018) chitosan coating incorporated with ethanolic extract of propolis prevented the oxidative deterioration of chicken fillets during cold storage and the PV was lower than control and chitosan groups without extract which is in agreement with our findings (Jonaidi Jafari et al., 2018). The effect of gelatin and chitosan coating on the quality of refrigerated rainbow trout fillets was evaluated by Nowzari et al. (2013). The PV of the fillets enhanced gradually in all samples during storage and significant differences were observed between samples with Gel/Ch and the control or pure Gel and pure Ch. Our results were in similarity to theirs that all coated fillets had lower PV than the control and shows that Gel/Ch coating could efficiently decrease lipid oxidation in fillets (Nowzari et al., 2013). It is demonstrated that chitosan, starch, and gelatin may be considered as the natural barriers against oxygen permeation and chitosan is presumed as a potential natural antioxidant for stabilizing lipid‐containing foods (Jonaidi Jafari et al., 2018; Perez Sira & Dufour, 2017).

**TABLE 11:** Peroxide value (meq/kg) of the chicken fillets treated with bitter orange extract and biocomposite coatings during storage at 4 ± 1°C.

	Time (day)				
	0	3	6	9	12
Control	0.01 ± 0.00^Aa^ *	0.22 ± 0.03^Ba^	0.59 ± 0.06^Ca^	0.76 ± 0.09^Da^	0.95 ± 0.10^Ea^
F1 (BOE)	0.01 ± 0.00^Aa^	0.09 ± 0.02^Bc^	0.15 ± 0.00^Cd^	0.28 ± 0.04^De^	0.38 ± 0.04^Ee^
F2 (Gel/MS)	0.01 ± 0.00^Aa^	0.20 ± 0.05^Ba^	0.35 ± 0.05^Cb^	0.57 ± 0.05^Db^	0.71 ± 0.11^Eb^
F3 (Gel/Ch)	0.01 ± 0.00^Aa^	0.14 ± 0.00^Bb^	0.29 ± 0.00^Cc^	0.45 ± 0.02^Dd^	0.61 ± 0.02^Ed^
F4 (MS/Ch)	0.01 ± 0.00^Aa^	0.19 ± 0.01^Ba^	0.38 ± 0.05^Cb^	0.52 ± 0.07^Dc^	0.67 ± 0.03^Ec^
F5 (Gel/MS/BOE)	0.01 ± 0.00^Aa^	0.08 ± 0.00^Ba^	0.12 ± 0.01^Ce^	0.25 ± 0.01^De^	0.38 ± 0.00^Ee^
F6 (Gel/Ch/BOE)	0.01 ± 0.00^Aa^	0.04 ± 0.00^Ad^	0.10 ± 0.02^Be^	0.22 ± 0.01^Cf^	0.34 ± 0.02^Df^
F7 (MS/Ch/BOE)	0.01 ± 0.00^Aa^	0.04 ± 0.00^Ad^	0.17 ± 0.02^Bd^	0.24 ± 0.00^Ce^	0.36 ± 0.05^De^

* Different letters in the columns (a–e) and in the rows (A–E) indicate statistically significant difference (*p* < .05). Data are presented as mean ± SD.

## 4 CONCLUSIONS

The pure extract of bitter orange peel had a considerable amount of phenolic compounds and expressed high antioxidant and antimicrobial activity in the chicken fillets. Combining BOE with biopolymer composites exerted higher preservative effect on the samples, increased BOE's activity, and also improved texture and drip loss. Fillets coated with biopolymer composites containing BOE showed considerably lower microbial count, FFA, PV, TVB‐N content, and drip loss throughout storage compared to the control, samples coated with pure BOE or biopolymer composite (without BOE). It should be noted that coatings containing chitosan had a higher antimicrobial and antioxidant effects. The coatings' activity as antimicrobial barriers and maintaining agents for physicochemical characteristics were as follows: Gel/Ch/BOE > MS/Ch/BOE > Gel/MS/BOE. It can be concluded that the applied composite coatings in this work have a high potential to maintain and improve the quality of raw chicken fillets during cold storage.

## AUTHOR CONTRIBUTIONS

**Maryam Azizkhani:** Conceptualization (equal); methodology (equal); project administration (equal); supervision (equal); validation (equal); visualization (equal); writing – original draft (equal); writing – review and editing (equal). **Sara Kavosi:** Conceptualization (equal); data curation (equal); formal analysis (equal); funding acquisition (equal); resources (equal); software (equal). **Razieh Partovi:** Conceptualization (equal); formal analysis (equal); software (equal).

## FUNDING INFORMATION

This work has been supported by a research grant from the Amol University of Special Modern Technologies, Amol, Iran.

## CONFLICT OF INTEREST STATEMENT

The authors declare that they do not have any conflict of interest.

## ETHICS STATEMENT

Not applicable.

## Data Availability

The data that support the findings of this study are available on request from the corresponding author.
